# Fixed-axis spacelike ruled surfaces and their evolute offsets

**DOI:** 10.1371/journal.pone.0325051

**Published:** 2025-06-06

**Authors:** Areej A. Almoneef, Rashad A. Abdel-Baky

**Affiliations:** 1 Department of Mathematical Sciences, College of Science, Princess Nourah Bint Abdulrahman University, Saudi Arabia; 2 Department of Mathematics, Faculty of Science, University of Assiut, Assiut, Egypt; Whale Wave Technology Inc., CHINA

## Abstract

This study investigates fixed-axis spacelike ruled surfaces and their evolute offset counterparts within $E_{1}^{3}$ (Minkowski 3-space). The analysis utilizes the Blaschke frame associated with the striction curves of these surfaces. Spacelike ruled surfaces play a crucial role in various fields of both classical and modern physics. The research begins by introducing the fundamental concepts of fixed-axis spacelike ruled surfaces and defining a height function that establishes the necessary criteria for a ruled surface to be classified as a fixed-axis spacelike ruled surface. Subsequently, the study derives parameterization for both the fixed-axis spacelike ruled surfaces and their evolute offsets. Finally, several surface models are extended and visually represented through graphical illustrations.

## 1 Introduction

In the context of movable frames, the most commonly used are the Frenet–Serret frame (SFF) for space curves and the Blaschke frame (BF) for ruled surfaces. The Blaschke frame is determined by the velocity of the striction curve and the normal vector of the associated sphere, whereas the Frenet–Serret frame is defined by the velocity and acceleration of the curve. By differentiating these movable frames with respect to their own basis vectors, certain real-valued functions emerge. These functions are known as curvature and torsion in the case of the Frenet–Serret frame and as the Blaschke invariants for the Blaschke frame (see, for example, [1–3]).

In the realm of line geometry, the set of oriented lines embedded in a moving solid body primarily generates ruled surfaces. The geometry of ruled surfaces has been extensively applied in various fields, including Computer-Aided Manufacturing (CAM), Computer-Aided Geometric Design (CAGD), geometric modeling, and motion analysis [3–7]. In recent years, the properties of ruled surfaces and their offset counterparts have been extensively studied in both Euclidean and non-Euclidean spaces. For example, Ravani and Ku [8] explored the theory of Bertrand curves for Bertrand ruled surface offsets using line geometry. They demonstrated that a ruled surface can possess an infinite number of Bertrand ruled surface offsets, similar to how a plane curve can have an infinite number of Bertrand mates. Building upon this work, Küçük and G ürsoy provided several examples of Bertrand offsets of trajectory ruled surfaces, analyzing their interrelations through projection domains and the corresponding spherical curve invariants [9]. In [10], Kasap and Kuruoğlu explored the relationships between the integral invariants of Bertrand ruled surfaces in Euclidean 3-space. In [11], they extended this research to the Bertrand offsets of ruled surfaces in $E_{1}^{3}$. The involute-evolute offsets of the ruled surfaces were studied by Kasap *et al*. in [12]. Orbay *et al*. [13] introduced the study of Mannheim offsets for ruled surfaces, while Önder and Uğurlu investigated the invariants of Mannheim offsets for timelike (TL) ruled surfaces and provided conditions for these surface offsets to be non-skew [14]. These offset surfaces are analyzed using the Blaschke frame, as defined in [8]. Based on the involute-evolute offsets of ruled surfaces in [12], Şentürk and Yüce computed the integral invariants of these offsets in relation to the geodesic SFF [15]. Recently, Yoon examined evolute offsets of ruled surfaces in both Euclidean and Minkowski 3-spaces, considering stationary Gaussian and mean curvatures [16, 17]. There is a substantial body of literature on these topics, including various treatises such as [18–20].

Subsequently, to consolidate interdisciplinary papers, we wish to highlight some significant studies on ruled surfaces and surface families in various spaces [21–23].

To the best of our knowledge, there has been no prior work on the construction of evolute offsets for a fixed-axis skew SL-ruled surface in $E_{1}^{3}$. This study aims to identify a set of invariants that describe the local shape of a fixed-axis SL -ruled surface and its evolute offsets. The conditions for two SL -ruled surfaces to be evolute offsets are developed, and the results are illustrated using computer-aided models. The findings presented in this paper offer valuable insight into surface theory, which could contribute to fields that require surface analysis.

## 2 Basic concepts

To meet the demands in the next sections, here, the crucial elements of the theory of curves in $E_{1}^{3}$ are briefly presented [1–4, 24, 25]. For vectors $𝔞=\left(a_{1},a_{2},a_{3}\right)$ and $𝔟=(b_{1},b_{2},b_{3})$, we know that


$$\left\langle 𝔞,𝔟\right\rangle =-a_{1}b_{1}+a_{2}b_{2}+a_{3}b_{3}\text{,}$$

is named Lorentzian inner product. The cross product produces a vector given by


$$𝔞\times 𝔳=(-a_{2}b_{3}+a_{3}b_{2},-a_{1}b_{3}+a_{3}b_{1},a_{1}b_{2}-a_{2}b_{1}).$$

Since ⟨,⟩ is an indefinite metric, recall that a vector $𝔞\in E_{1}^{3}$ can possess one of three causal natures; it can be SL if ⟨a,𝔞⟩>0 or 𝔞=0, TL if ⟨𝔞,𝔞⟩<0 and null or lightlike if ⟨𝔞,𝔞⟩=0 and 𝔞≠0. The norm of $𝔞\in E_{1}^{3}$ is pointed by $\left\|𝔞\right\|=\sqrt{\left|\left\langle 𝔞,𝔞\right\rangle \right|}$, then the hyperbolic and Lorentzian (de Sitter space) unit spheres are:

$${ℋ}_{+}^{2}=\{𝔞\in E_{1}^{3}|{\left\|𝔞\right\|}^{2}:=-a_{1}^{2}+a_{2}^{2}+a_{3}^{2}=-1\},$$ (1)

and

$${𝒮}_{1}^{2}=\{𝔞\in E_{1}^{3}|{\left\|𝔞\right\|}^{2}:=-a_{1}^{2}+a_{2}^{2}+a_{3}^{2}=1\}.$$ (2)

A SL line $ℒ\in E^{3}$ can be attended by a point 𝔭∈ℒ and a TL unit vector 𝔞 on it, that is, ${\left\|𝔞\right\|}^{2}=1$. A parametric equation of ℒ is

ℒ: 𝔮=𝔭+t𝔞; t∈ℝ. (3)

Thus, we set the moment ${𝔞}^{*}$ with reference to a fixed origin point as

$${𝔞}^{*}=𝔮\times 𝔞=𝔭\times 𝔞,$$ (4)

where

$${\left\|𝔞\right\|}^{2}=1,\;<𝔞,{𝔞}^{*}>=0.$$ (5)

Wherefore, we can write that $ℒ=\left(𝔞,{𝔞}^{*}\right)\in E_{1}^{3}\times E_{1}^{3}$. Let ${ℒ}_{i}=\left({𝔞}_{i},{𝔞}_{i}^{*}\right)$ be two SL lines assigned with ${𝔞}_{1}$ and ${𝔞}_{2}$ linearly independent. Then the distance $\beta^{*}({ℒ}_{1},{ℒ}_{2})$ among ${ℒ}_{1}$, ${ℒ}_{2}$ is

$$\beta^{*}({ℒ}_{1},{ℒ}_{2})=\mp \frac{\left(<a_{1},{𝔞}_{2}^{*}>+<{𝔞}_{2},{𝔞}_{1}^{*}>\right)}{\left\|{𝔞}_{1}\times {𝔞}_{2}\right\|}.$$ (6)

The Lorentzian distance in the customary sense will then be $\left|\beta^{*}({ℒ}_{1},{ℒ}_{2})\right|$. The angle among ${ℒ}_{1}$, ${ℒ}_{2}$ is specified as follows:

1) If they span a SL plane; there is a unique angle ϑ ; 0≤ϑ≤π such that

$$\beta ({ℒ}_{1},{ℒ}_{2})=\cos^{-1}<{𝔞}_{1},{𝔞}_{2}>,$$ (7)

2) If they span a TL plane, there is a unique angle ϑ ; ϑ≥0 such that

$$\beta ({ℒ}_{1},{ℒ}_{2})=\cosh^{-1}<{𝔞}_{1},{𝔞}_{2}>.$$ (8)

The spatial distance between ${ℒ}_{1}$, ${ℒ}_{2}$ is located to be a relationship of real numbers.

$$spd({ℒ}_{1},{ℒ}_{2})=\left(\left|\beta^{*}({ℒ}_{1},{ℒ}_{2})\right|,\beta ({ℒ}_{1},L_{2})\right).$$ (9)

### 2.1 Ruled surface

A ruled surface is a surface generated by a line ℒ that moves along a curve 𝔷(v). The various positions of these lines are referred to as the generators of the surface. Such a surface has the parametric representation [1, 2 , 20, 21]:

𝔐:𝔶(v,t)=𝔷(v)+t𝔪(v),t∈I,v∈ℝ, (10)

where ‖𝔪‖2=σ(±1), ‖𝔪′‖2=η(±1), <𝔷′,𝔪′>=0; $\prime =\frac{d}{dv}$. In this setting, the curve 𝔷(v) is known as the striction curve, and *v* is the arc-length of $𝔪(v)\in {𝒮}_{1}^{2}$ (or ${ℋ}_{+}^{2}$). If 𝔪 is neither stationary nor null, and if ${𝔪}^{\prime }$ is non-null, then the Blaschke frame for 𝔪(v) can be established as follows [20, 24]:


$$\begin{array}{c} {m=m}_{1}(v),\;{𝔪}_{2}(v)={𝔪}^{\prime },\;{𝔪}_{3}={𝔪}_{1}\times {𝔪}_{2},\\ {𝔪}_{1}\times {𝔪}_{2}={𝔪}_{3},\;\;{𝔪}_{1}\times {𝔪}_{3}=\sigma {𝔪}_{2},\;\;{𝔪}_{2}\times {𝔪}_{3}=-\eta {𝔪}_{1},\;{\left\|{𝔪}_{3}\right\|}^{2}=-\sigma \eta . \end{array}\}$$

This provides a compact framework for analyzing the geometric properties of the ruled surface using the Blaschke frame. The Blaschke formula for the Blaschke frame is expressed as:


$$\left[\begin{array}{c} {𝔪}_{1}^{\prime }\\ {𝔪}_{2}^{\prime }\\ {𝔪}_{3}^{\prime } \end{array}\right]=\left[\begin{array}{ccc} 0 & 1 & 0\\ -\sigma \eta & 0 & J\\ 0 & \sigma J & 0 \end{array}\right]\left[\begin{array}{c} {𝔪}_{1}\\ {𝔪}_{2}\\ {𝔪}_{3} \end{array}\right],$$

where $J(v)=\det({𝔪}^{\prime \prime },{𝔪}^{\prime },𝔪)$ represents the spherical curvature of 𝔪(v). With respect to the Blaschke frame and the signs σ, η, −ση, the striction curve is defined as [20, 24]:

$${𝔷}^{\prime }(v)=\overset{v}{\underset{0}{\int }}(\sigma \lambda (v){𝔪}_{1}(v)-\sigma \eta \mu (v){𝔪}_{3}(v))dv,$$ (11)

Here, *J*(*v*), λ(v) and μ(v) are known as the curvature functions of 𝔐. This formulation provides a systematic approach to describing the geometry of ruled surfaces in terms of their Blaschke frame components and curvature functions.

## 3 Main results

In this section, we explore and define the evolute offsets of a skew fixed-axis SL-ruled surface in $E_{1}^{3}$, utilizing the symmetry of the evolute curves. We then provide the parameterization of the evolute offsets for both skew and non-skew fixed-axis SL -ruled surfaces. Furthermore, we examine the properties of these ruled surfaces and discuss a classification scheme for their various forms.

Based on the notations introduced in Section 2, we focus on a skew SL-ruled surface characterized by (σ,η)=(1,−1). From this, we derive the following results:


𝔐:𝔶(v,t)=𝔷(v)+t𝔪(v),t∈I,v∈ℝ,

where


‖𝔪‖2=−‖𝔪′‖2=1,<𝔷′,𝔪′>=0,′=ddv,

Then, the Blaschke formula is

$$\left[\begin{array}{c} {𝔪}_{1}^{\prime }\\ {𝔪}_{2}^{\prime }\\ {𝔪}_{3}^{\prime } \end{array}\right]=\left[\begin{array}{ccc} 0 & 1 & 0\\ 1 & 0 & J\\ 0 & J & 0 \end{array}\right]\left[\begin{array}{c} {𝔪}_{1}\\ {𝔪}_{2}\\ {𝔪}_{3} \end{array}\right]=\varpi \times \left[\begin{array}{c} {𝔪}_{1}\\ {𝔪}_{2}\\ {𝔪}_{3} \end{array}\right],$$ (12)

where $\varpi (v)=J(v){𝔪}_{1}(v{)-m}_{3}(v)$ is the Darboux vector, and

$$\begin{array}{c} {𝔪}_{1}\times {𝔪}_{2}={𝔪}_{3},\;\;{𝔪}_{1}\times {𝔪}_{3}={𝔪}_{2},\;{𝔪}_{2}\times {𝔪}_{3}={𝔪}_{1},\;\\ {\left\|{𝔪}_{1}\right\|}^{2}=-{\left\|{𝔪}_{2}\right\|}^{2}={\left\|{𝔪}_{3}\right\|}^{2}=1. \end{array}\}$$ (13)

The striction curve is defined by

$$𝔷(v)=\overset{v}{\underset{0}{\int }}(-\lambda (v){𝔪}_{1}(v)+\mu (v){𝔪}_{3}(v))dv.$$ (14)

Therefore, a skew SL-ruled surface can be described as:

$$𝔐:𝔶(v,t)=𝔷(v)+t{𝔪}_{1}(v),\;t\in I,\;v\in \mathbb{R}.$$ (15)

*J*(*v*), μ(v) and λ(v) are the structure functions of 𝔐; *J*(*v*) is the spherical curvature of $𝔪(v)\in {𝒮}_{1}^{2}$, $\lambda (v)=\cos^{-1}<{𝔷}^{\prime }(v),{𝔪}_{1}>$ and μ(v) is the distribution parameter of M.

**Definition 1.** 𝔐* is a fixed-angle SLRS if its ruling has a fixed-angle with a definite line.*

**Definition 2.** 𝔐* is a fixed-distance SLRS if its ruling has a fixed-distance with a definite line.*

**Definition 3.** 𝔐* is a fixed-axis SLRS if its ruling has a fixed-spatial distance with a definite line.*

Furthermore, the SL curvature-axis of ${𝔪}_{1}(v)\in {𝒮}_{1}^{2}$ is

$$𝔤(v)=\frac{\varpi }{\left\|\varpi \right\|}=\frac{J}{\sqrt{J^{2}+1}}{𝔪}_{1}+\frac{1}{\sqrt{J^{2}+1}}{𝔪}_{3}.$$ (16)

Let β be the radius of curvature among ${𝔪}_{1}$ and 𝔢. Then

$$𝔤(v)=\cos\beta {𝔪}_{1}+\sin\beta {𝔪}_{3}\text{ , with }\cot\beta =J(v).$$ (17)

**Corollary 1.** *The curvature κ(v), the torsion τ(v), and J(v) of ${𝔪}_{1}(v)\in {𝒮}_{1}^{2}$ are*

κ(v)=J2+1=1sinβ=1ρ(v),τ(v):=±β′=±J′J2+1. (18)

**Corollary 2.** *If J(v)=const., then ${𝔪}_{1}(v)\in {𝒮}_{1}^{2}$ is a Lorentzian small circle.*

**Proof**. From Eq 18, we observe that J=const.⇒τ(v)=0, and κ(v)=const., which indicates that ${𝔪}_{1}(v)\in {𝒮}_{1}^{2}$ is a Lorentzian small circle (assuming J(v)≠0) ∎.

**Definition 4.** *Let 𝔐 be a skew SLRS that satisfies* Eq 15 *in $E_{1}^{3}$. A RS ${𝔐}^{*}$ is an evolute offset of 𝔐 if there exists a bijection between their striction points such that the central normal of 𝔐 and the ruling of ${𝔐}^{*}$ are colinear.*

Let ${𝔐}^{*}$ be an evolute offset of 𝔐. Then,

$${𝔐}^{*}:{𝔶}^{*}(v,t)={𝔷}^{*}(v)+t{𝔪}_{2}(v),\;t\in \mathbb{R},$$ (19)

where

$${𝔷}^{*}(v)=𝔷(v)+f(v){𝔪}_{2}(v).$$ (20)

Here *f*(*v*) is the distance function among the striction points of 𝔐 and ${𝔐}^{*}$ [16, 17]. If ${𝔪}_{1}^{*}$, ${𝔪}_{2}^{*}$ and ${𝔪}_{3}^{*}$ are the Blaschke vectors of ${𝔐}^{*}$, and since ${𝔪}_{1}^{*}={𝔪}_{2}$ at striction points, then,

$${𝔪}_{2}^{*}:=\frac{{𝔪}_{2}^{\prime }}{\left\|{𝔪}_{2}^{\prime }\right\|}=\frac{1}{\sqrt{J^{2}+1}}m_{1}+\frac{J}{\sqrt{J^{2}+1}}{𝔪}_{3},$$ (21)

and

$${𝔪}_{3}^{*}:={𝔪}_{1}^{*}\times {𝔪}_{2}^{*}=\frac{J}{\sqrt{J^{2}+1}}{𝔪}_{1}-\frac{1}{\sqrt{J^{2}+1}}{𝔪}_{3}.$$ (22)

In view of Eqs 17, (21), and (22) we reach

$$\left[\begin{array}{c} {𝔪}_{1}^{*}\\ {𝔪}_{2}^{*}\\ {𝔪}_{3}^{*} \end{array}\right]=\left[\begin{array}{ccc} 0 & 1 & 0\\ \sin\beta & 0 & \cos\beta \\ \cos\beta & 0 & -\sin\beta \end{array}\right]\left[\begin{array}{c} {𝔪}_{1}\\ {𝔪}_{2}\\ {𝔪}_{3} \end{array}\right].$$ (23)

where


$${𝔪}_{1}^{*}\times {𝔪}_{2}^{*}={𝔪}_{3}^{*},\;{𝔪}_{1}^{*}\times {𝔪}_{3}^{*}=-{𝔪}_{2}^{*},\;\;{𝔪}_{2}^{*}\times {𝔪}_{3}^{*}=-{𝔪}_{1}^{*}.$$

If ${\text{v}}^{*}$ be the arc-length of ${𝔪}_{1}^{*}\in {ℋ}_{+}^{2}$, then $dv^{*}:=\left\|{𝔪}_{2}^{\prime }\right\|dv=\sqrt{J^{2}+1}dv$. Therefore,

$$\frac{d}{dv^{*}}\left[\begin{array}{c} {𝔪}_{1}^{*}\\ {𝔪}_{2}^{*}\\ {𝔪}_{3}^{*} \end{array}\right]=\left[\begin{array}{ccc} 0 & 1 & 0\\ 1 & 0 & J^{*}\\ 0 & -J^{*} & 0 \end{array}\right]\left[\begin{array}{c} {𝔪}_{1}^{*}\\ {𝔪}_{2}^{*}\\ {𝔪}_{3}^{*} \end{array}\right],$$ (24)

where

J*(v)=±J′(v)(J2(v)+1)32. (25)

**Corollary 3.** J(v)=const.*, that is, ${𝔪}_{1}(v)\in {𝒮}_{1}^{2}$ is a Lorentzian small circle iff $J^{*}(v)=0$, that is, ${𝔪}_{1}^{*}(v^{*})\in {ℋ}_{+}^{2}$ is a hyperbolic great circle.*

### 3.1 Height functions

In matching with [26], a point ${𝔤}_{0}\in {𝒮}_{1}^{2}$ will be ${𝔤}_{p}$ curvature-axis of$\;𝔪(v)\in {𝒮}_{1}^{2}$; for all *v* such that $<{𝔤}_{0},{𝔪}^{p}(v)>=0$, with $<{𝔤}_{0},{𝔪}^{p+1}(v)>\neq 0$. Here ${𝔪}^{p+1}$ signalizes the *p*-th derivative of 𝔪(v) with reference to *v*. For the 1st curvature-axis 𝔤 of$\;𝔪(v)\in {ℋ}_{+}^{2}$, we locate $<𝔤,{𝔪}_{1}^{\prime }>=\pm <𝔤,{𝔪}_{2}>=0$, and $<𝔤,{𝔪}_{1}^{\prime \prime }>=\pm <𝔤,{𝔪}_{1}+J{𝔪}_{3}>\neq 0$. So, 𝔤 is at least a ${𝔤}_{2}$ curvature-axis of $𝔪(v)\in {𝒮}_{1}^{2}$. We now locate a high function $w:I\times {𝒮}_{1}^{2}\to \mathbb{R}$, by $w(v,{𝔤}_{0})=<{𝔤}_{0},{𝔪}_{1}>$. We let $w(v)=w(v,{𝔤}_{0})$ for any constant point ${𝔤}_{0}\in {𝒮}_{1}^{2}$. Then, we display the following:

**Proposition 1.** *Via the last presuppositions, we occupancy:*

*i- w is fixed up to the 1st order iff ${𝔤}_{0}\in Sp\{{𝔪}_{1}$,${𝔪}_{3}\}$, that is,*


$$w^{\prime }=0\Leftrightarrow <{𝔪}_{1}^{\prime },{𝔤}_{0}>=0\Leftrightarrow <{𝔪}_{2},{𝔤}_{0}>=0\Leftrightarrow {𝔤}_{0}=c_{1}{𝔪}_{1}+c_{3}{𝔪}_{3};$$

*for $c_{1},c_{3}\in \mathbb{R},$ and $c_{1}^{2}+c_{3}^{2}=1$.*

*ii- w is fixed up to the 2nd order iff ${𝔤}_{0}$ is ${𝔤}_{2}$ curvature-axis of $𝔪(v)\in {𝒮}_{1}^{2}$, that is,*


$$w^{\prime }=w^{\prime \prime }=0\Leftrightarrow {𝔤}_{0}=\pm 𝔤.$$

*iii- w is fixed up to the 3rd iff ${𝔤}_{0}$ is ${𝔤}_{3}$ curvature-axis of $𝔪(v)\in {𝒮}_{1}^{2}$, that is,*


$$w^{\prime }=w^{\prime \prime }=w^{\prime \prime \prime }=0\Leftrightarrow {𝔤}_{0}=\pm 𝔤,\;and\;J^{\prime }\neq 0.$$

*iv- w is fixed up to the 4th order iff ${𝔤}_{0}$ is ${𝔤}_{4}$ curvature-axis of $𝔪(v)\in {𝒮}_{1}^{2}$, that is,*


$$w^{\prime }=w^{\prime \prime }=w^{\prime \prime \prime }=w^{iv}=0\Leftrightarrow {𝔤}_{0}=\pm 𝔤\text{, }J^{\prime }=0,\;and\;J^{\prime \prime }\neq 0.$$

**Proof**. i-Firstly, we derive

$$w^{\prime }=<{𝔪}_{1}^{\prime },{𝔤}_{0}>.$$ (26)

Then,

$$w^{\prime }=0\Leftrightarrow <{𝔪}_{2},{𝔤}_{0}>=0\Leftrightarrow {𝔤}_{0}=c_{1}{𝔪}_{1}+c_{3}{𝔪}_{3};$$ (27)

for real numbers *c*_1_, $c_{3}\in \mathbb{R},$ and $c_{1}^{2}+c_{3}^{2}=1$, the consequence is apparent.

ii- The derivative of Eq 26 register that:

$$w^{\prime \prime }=<{𝔪}_{1}^{\prime \prime },{𝔤}_{0}>=<{𝔪}_{1}+J{𝔪}_{3},{𝔤}_{0}>.$$ (28)

By Eqs 26, and (28) we attain


$$w^{\prime }=w^{\prime \prime }=0\Leftrightarrow <{𝔪}_{1}^{\prime },{𝔤}_{0}>=<{𝔪}_{1}^{\prime \prime },{𝔤}_{0}>=0\Leftrightarrow {𝔤}_{0}=\pm \frac{{𝔤}^{\prime }\times {𝔤}^{\prime \prime }}{\left\|{𝔤}^{\prime }\times {𝔤}^{\prime \prime }\right\|}=\pm 𝔤.$$

iii- The derivative of Eq 28 is


w′′′=<𝔪1′′′,𝔤0>=(1+J2)<𝔪2,𝔤0>+J′<𝔪3,𝔤0>.

Hence, we attain


w′=w′′=w′′′=0⇔𝔤0=±𝔤, and J′≠0.

iv- By the same pretexts, we can also control


w′=w′′=w′′′=w′′′′=0⇔𝔤0=±𝔤, J′=0, and J′′≠0.

The proof is complete ∎.

Via the Proposition 1, we conclude:

(a) The osculating circle (OS) $𝒮(\rho ,{𝔤}_{0})$ of $𝔪(v)\in {𝒮}_{1}^{2}$ is width by


<𝔤0,𝔪1>=ρ(v),<𝔪1′,𝔤0>=0,<𝔪1′′,𝔤0>=0,

which state that the OS must has link of at least 3rd order at $𝔤(v_{0})$ iff J^′′^≠0.

(b) The curve $𝔪(v)\in {𝒮}_{1}^{2}$ and the OS $𝒮(\rho ,{𝔤}_{0})$ has link at least 4-th order at $𝔤(v_{0})$ iff J^′^=0, and J^′′^≠0.

In this track, by taking into examination the curvature-axes of $𝔪(v)\in {𝒮}_{1}^{2}$, we can accomplish a sequence of curvature-axes ${𝔤}_{2}$, ${𝔤}_{3}$,..., ${𝔤}_{n}$. The proprietorships and the joint links among these curvature-axes are much amusing topics. For demand, it is uncomplicated to see that if ${𝔤}_{0}=\pm 𝔤$, and J^′^=0, 𝔪(v) is locating at β is fixed regarding to ${𝔤}_{0}$. In these circumstances, the curvature axis is fixed up to 2nd order, and 𝔐 is a constant angle SLRS.

**Theorem 1.** 𝔐* is a fixed-angle SLRS, that is, β(v)=const iff J(v)=const.*

Since J(v)=const., from the Eqs 12, and (18), we reach to: ${𝔪}_{1}^{\prime \prime \prime }-\kappa^{2}{𝔪}_{1}^{\prime }=0$. It is valuable to transfer the parameter *v* via $d\varphi =\sqrt{J^{2}+1}dv$, and let’s take ${𝔪}_{1}^{\prime }(\varphi )=\left(0,1,0\right)$. Then,


$${𝔪}_{1}^{\prime }(\varphi )=\left(a_{1}\sinh\varphi ,\cosh\varphi +a_{2}\sinh\varphi ,a_{3}\sinh\varphi \right),$$

where *a*_1_, *a*_2_, *a*_3_ are constants fulfilling *a*_2_ = 0, and $a_{2}^{2}+a_{3}^{2}=1$. It follows that

$${𝔪}_{1}(\varphi )=\left(a_{1}\sin\beta \cosh\varphi +b_{1},\sin\beta \sinh\varphi ,a_{3}\sin\beta \cosh\varphi +b_{3}\right),$$ (29)

for constants *a*_2_, *a*_3_ fulfilling $a_{1}b_{1}+a_{3}b_{3}=0$, and $b_{1}^{2}+b_{3}^{2}=\cos^{2}\beta$. Let’s make


$$\left[\begin{array}{c} x_{1}\\ x_{2}\\ x_{3} \end{array}\right]=\left[\begin{array}{ccc} a_{1} & 0 & a_{3}\\ 0 & 1 & 0\\ -a_{3} & 0 & a_{1} \end{array}\right]\left[\begin{array}{c} x\\ y\\ z \end{array}\right].$$

Therefore, we attain


$${𝔪}_{1}(\varphi )=\left(\sin\beta \cosh\varphi ,,\sin\beta \sinh\varphi ,a_{1}b_{3}-a_{3}b_{1}\right).$$

Since ${\left\|{𝔪}_{1}\right\|}^{2}=1$, we realize that $a_{1}b_{3}-a_{3}b_{1}=\pm \cos\beta$. By setting the upper sign, we receive

$${𝔪}_{1}(\varphi )=\left(\sin\beta \cosh\varphi ,\sin\beta \sinh\varphi ,\cos\beta \right).$$ (30)

Then,

$$\begin{array}{c} {𝔪}_{2}(\varphi )=\frac{d{𝔪}_{1}}{d\varphi }{\left\|\frac{d{𝔪}_{1}}{d\varphi }\right\|}^{-1}=\left(\sinh\varphi ,\cosh\varphi ,0\right),\\ {𝔪}_{3}(\varphi )={𝔪}_{1}\times {𝔪}_{2}=\left(\cos\beta \cosh\varphi ,\cos\beta \sinh\varphi ,\sin\beta \right). \end{array}\}$$ (31)

Let

$$𝔷(v)=z_{1}(v){𝔪}_{1}(v)+z_{2}(v){𝔪}_{2}(v)+z_{3}(v){𝔪}_{3}(v),$$ (32)

where $z_{1}(v),\;z_{2}(v)\;$and $z_{3}(v)$ are differentiable functions of v∈I⊆ℝ. Differentiating the last equation via *v* and appointing the Blaschke formulae, we possess

$${𝔷}^{\prime }(v)=(z_{1}^{\prime }+z_{2}){𝔪}_{1}+(z_{2}^{\prime }+Jz_{3}+z_{1}){𝔪}_{2}+(z_{3}^{\prime }+Jz_{2}){𝔪}_{3}.$$ (33)

From Eqs 14, and (33), one finds that:

$$\begin{array}{c} z_{1}^{\prime }+z_{2}=-\lambda ,\\ z_{2}^{\prime }+Jz_{3}+z_{1}=0,\\ z_{3}^{\prime }+Jz_{2}=\mu , \end{array}\}$$ (34)

which signify we can manifest that

$$\frac{d^{2}z_{2}}{d\varphi^{2}}-z_{2}=\frac{\lambda +J\mu }{J^{2}+1}.$$ (35)

## 4 Evolute offsets of a fixed-axis SLSL-ruled surface

In this section, we conclude and inspect the evolute offsets of a constant-axis skew and non-skew SL-ruled surfaces. We then develop a theory analogous to the theory of evolute curves for these surfaces. To achieve this, we present the following theorem.

**Theorem 2.** *Let 𝔐 be a skew SL-ruled surface as defined in* Eq 15*. Then 𝔐 is a fixed-axis SL-ruled surface iff (i) *J* = *const*., and (ii) λ+Jμ=const.*

**Fig 1:**
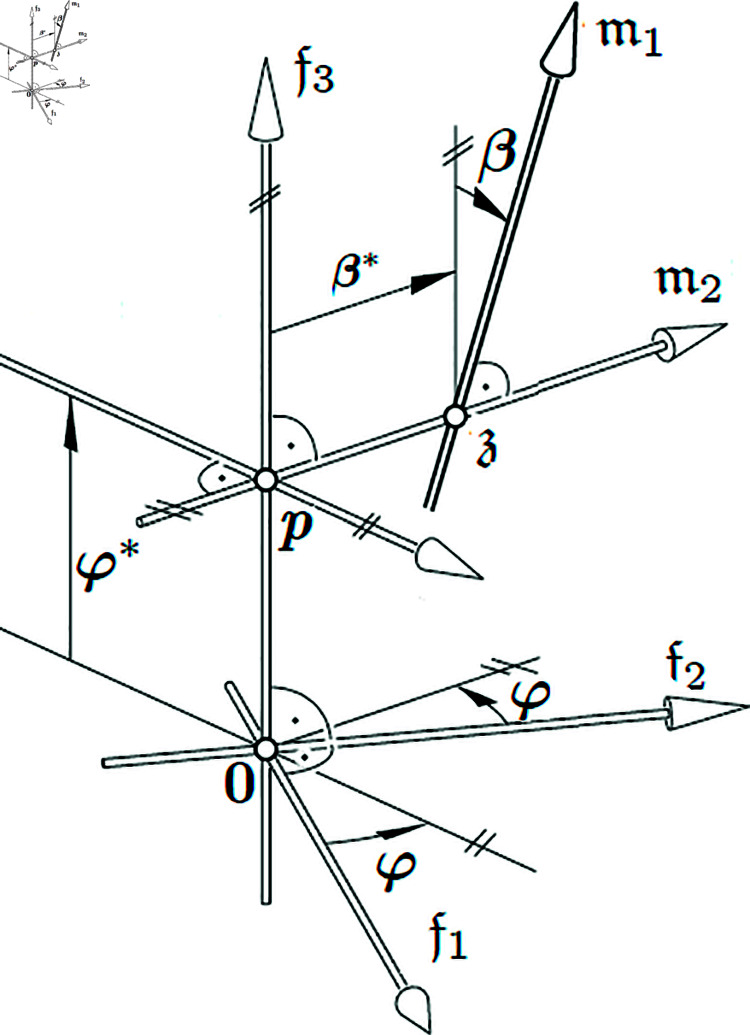
A special Lorentzian fixed frame.

**Proof**. The necessity of the conditions follows directly from Theorem 1. For the sufficiency, we proceed as follows: Without loss of generality, a constant Lorentzian frame $\left\{0;\;{𝔣}_{1},{𝔣}_{2},\;{𝔣}_{3}\right\}$ can be used with the ${𝔣}_{3}(=𝔤(\varphi )$). The striction curve can be determined by


$$𝔷(\varphi )=\varphi^{*}(\varphi ){𝔣}_{3}+\beta^{*}{𝔪}_{2}(\varphi ),$$

or in view of Eqs 31, we attain

$$𝔷(\varphi )=\beta^{*}(\varphi )\sinh\varphi {𝔣}_{1}+\beta^{*}(\varphi )\cosh\varphi {𝔣}_{2}+\varphi^{*}(\varphi ){𝔣}_{3},$$ (36)

where $\varphi^{*}$ is the distance along the ${𝔣}_{3}-$axis (Figure 1). The stations of both points p and 𝔷 determined on that of ${𝔪}_{1}$; the minimal distance $\beta^{*}$ is based on $<{𝔪}_{1},{𝔣}_{3}>$.

From Eqs 30-(32) and Eq 36 we acquire

$$\varphi^{*}:=z_{1}\cos\beta +z_{3}\sin\beta ,\;\;z_{2}=\beta^{*}.$$ (37)

If $\beta^{*}(=z_{2})=const$, then Eq 27 leads to

$$\lambda +J\mu =-(J^{2}+1)\beta^{*}\Rightarrow \lambda +J\mu =const.$$ (38)

The proof is done ∎.

Since $\beta^{*}$ and β are all constants, this exhibits that 𝔷(φ) is a cylindrical helix with the ${𝔣}_{3}$ -axis. Furthermore, for $z_{2}^{\prime }=0$, from Eqs 18, and (38), we accomplish

$$z_{2}(=\beta^{*}):=-\frac{\lambda +J\mu }{J^{2}+1}=(\lambda \sin\beta +\mu \cos\beta )\sin\beta .$$ (39)

In view of Eqs 34, and (39), we fulfill

$$\begin{array}{c} z_{1}^{\prime }=(\lambda \cos\beta -\mu \sin\beta )\cos\beta ,\\ z_{3}^{\prime }=-(\lambda \cos\beta -\mu \sin\beta )\sin\beta . \end{array}\}$$ (40)

Hence, from Eqs 15, (30), (39), and (40), we attain

$$𝔐:𝔶(\varphi ,t)=(z_{1}+t\sin\beta \cosh\varphi ,z_{2}+t\sin\beta \sinh\varphi ,z_{3}+t\cos\beta ),$$ (41)

where

$$\begin{array}{c} z_{1}=[(\overset{\varphi }{\underset{0}{\int }}\lambda d\varphi )\cos\beta -(\overset{\varphi }{\underset{0}{\int }}\mu d\varphi )\sin\beta ]\cos\beta ,\\ z_{2}=(\lambda \sin\beta +\mu \cos\beta )\sin\beta ,\\ z_{3}=-[(\overset{\varphi }{\underset{0}{\int }}\lambda d\varphi )\cos\beta -(\overset{\varphi }{\underset{0}{\int }}\mu d\varphi )\sin\beta ]\sin\beta . \end{array}\}$$ (42)

Via Eqs 19, and (41) the surface ${𝔐}^{*}$ is

$${𝔐}^{*}:\;{𝔶}^{*}(\varphi ,t)=\left[\begin{array}{c} z_{1}+(f(\varphi )+t)\sinh\varphi \\ z_{2}+(f(\varphi )+t)\cosh\varphi \\ z_{3} \end{array}\right],\;t\in \mathbb{R}.$$ (43)

### 4.1 Classification of 𝔐M and ${𝔐}^{*}$M

**Fig 2:**
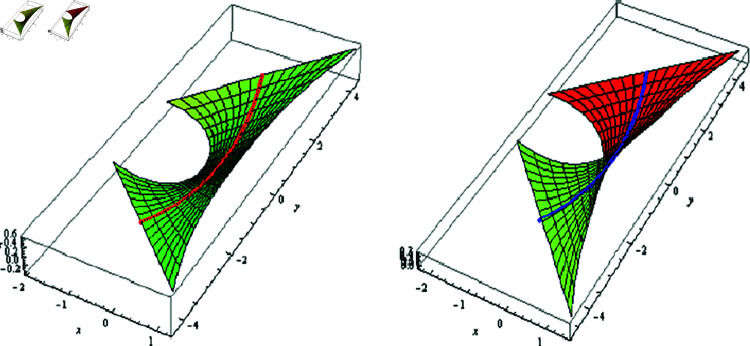
𝔐 (left) and its evolute offset ${𝔐}^{*}$ (right), f(φ)=−0.005coshφ,λ(φ)=μ(φ)=φ

**Fig 3:**
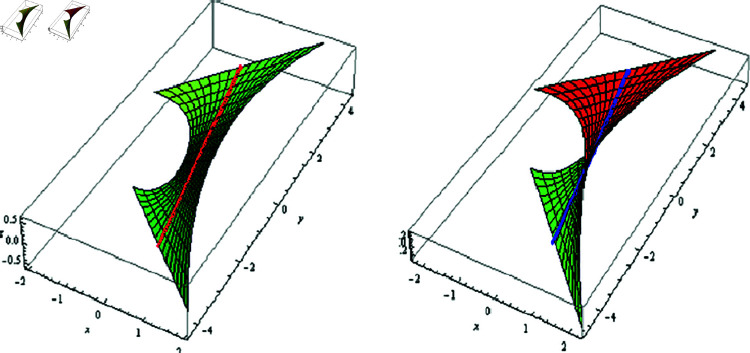
𝔐 (left) and its evolute offset ${𝔐}^{*}$ (right), f(φ)=−0.005coshφ, λ(φ)=μ(φ)=1

**Fig 4:**
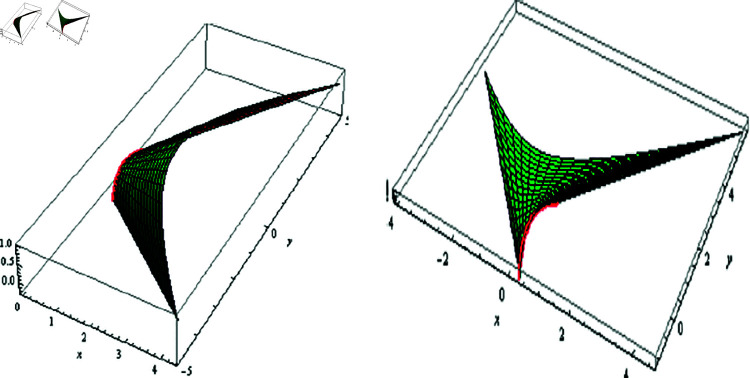
𝔐 (left) and its evolute offset ${𝔐}^{*}$ (right), f(φ)=−0.005coshφ, λ=φ,μ=0

**Fig 5:**
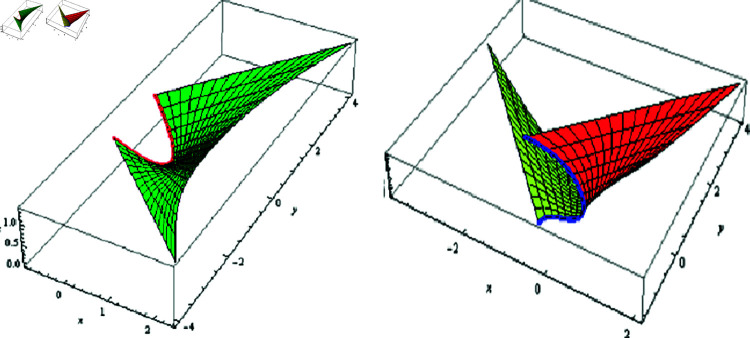
𝔐 (left) and its evolute offset ${𝔐}^{*}$ (right), f(φ)=−0.005coshφ, λ=0,μ=φ

In the following, we set β=π/3. For specific values of λ(φ), μ(φ), and 𝔣(φ), we consider the following:

(1) Let λ(φ)=μ(φ)=φ, f(φ)=−0.005coshφ, −0.5≤t≤0.5, and −2≤φ≤2. The fixed-axis SLRS, and its evolute offset appear in Fig 2.

(2) Let λ(φ)=μ(φ)=1, f(φ)=−0.005coshφ, −0.5≤t≤0.5, and −2≤φ≤2. The fixed-axis SLRS, and its evolute offset appear in Fig 3.

(3) Since μ=0, then 𝔐 is an SL non-skew or tangential developable (TD). For λ=φ, f(φ)=−0.005coshφ, 0≤t≤2, and −1.5≤φ≤1.5, the fixed-axis 𝔐, and its evolute offset ${𝔐}^{*}$ are located in Fig 4.

(4) Since λ=0, then 𝔐 is a SL binormal (ℬ). For μ=φ, f(φ)=−0.005coshφ, 0≤t≤1, and −2≤φ≤2, the fixed-axis 𝔐, and its evolute offset ${𝔐}^{*}$ are located in Fig 5.

(5) If 𝔐 is a SL cone, then $𝔷(\varphi {)}^{\prime }=0$. From Eq 36, we conclude

$$\beta^{*}\cosh\varphi {𝔣}_{1}+\beta^{*}\sinh\varphi {𝔣}_{2}+\varphi^{{*}^{\prime }}(\varphi ){𝔣}_{3}=0.$$ (44)

Then,

$$\beta^{*}\cosh\varphi =0\text{, }\beta^{*}\sinh\varphi =0\text{, }\varphi^{{*}^{\prime }}=0,$$ (45)

which show that $\beta^{*}=0$, and $\varphi^{*}=const$. Further, employing $\beta^{*}=0$ into the Eq 39 we deduce λ(φ)=μ(φ)=0. Consequently, we acquire

ℜ:𝔶(φ,t)=t(sinβcoshφ,sinβsinhφ,cosβ), (46)

and

$${𝔐}^{*}:\;{𝔶}^{*}(\varphi ,t)=((f(\varphi )+t)\sinh\varphi ,(f(\varphi )+t)\cosh\varphi ,0).$$ (47)

**Fig 6:**
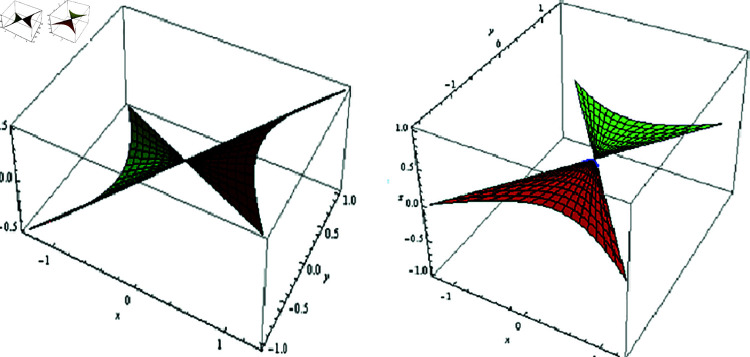
𝔐 (left) and its evolute offset ${𝔐}^{*}$ (right), f(φ)=−0.005coshφ, λ=μ=0.

The fixed-axis 𝔐, and its evolute offset ${𝔐}^{*}$ are arranged in Fig 6; where f(φ)=−0.005coshφ, −1≤t≤1 and −1≤φ≤1.

## 5 Conclusion

In this study, we examined the evolute offsets of fixed-axis skew SL-ruled surface in $E_{1}^{3}$, by leveraging the symmetry of evolute curves. Through our analysis, we formulated the parameterization of evolute offsets for both skew and non-skew ruled surfaces while also characterizing their geometric properties. We established key structural relationships between the curvature-axis functions and the associated height functions, leading to a deeper understanding of the curvature behavior of skew SL-ruled surface. In particular, we derived conditions under which a skew SL-ruled surface becomes a fixed-axis SL -ruled surface and formulated a theorem analogous to the classical theory of evolute curves, providing a framework for further studies on these ruled surfaces.

Our results highlight that a surface 𝔐 is a fixed-axis SL-ruled surface if and only if the spherical curvature function *J* remains constant and the function λ+Jμ is also constant. These findings contribute to the broader study of ruled surfaces in Lorentzian geometry and offer a foundation for potential applications in kinematics and differential geometry. Future research may explore generalizations of these results in higher-dimensional spaces or in relation to other classes of ruled surfaces.

These results are expected to be useful in the field of CAGD/CAM. In future work, we plan to further investigate the classification of singularities as outlined in [27, 28].
